# A Nanopore Sequencing-based Pharmacogenomic Panel to Personalize Tuberculosis Drug Dosing

**DOI:** 10.1164/rccm.202309-1583OC

**Published:** 2024-03-05

**Authors:** Renu Verma, Kesia Esther da Silva, Neesha Rockwood, Roeland E. Wasmann, Nombuso Yende, Taeksun Song, Eugene Kim, Paolo Denti, Robert J. Wilkinson, Jason R. Andrews

**Affiliations:** ^1^ Division of Infectious Diseases and Geographic Medicine, Stanford University School of Medicine, Stanford, California;; ^2^ Manipal Academy of Higher Education, Manipal, India;; ^3^ Institute of Bioinformatics, International Tech Park, Bangalore, India;; ^4^ Wellcome Center for Infectious Diseases Research in Africa, Institute of Infectious Disease and Molecular Medicine and Department Medicine,; ^5^ Department of Pathology and Institute of Infectious Disease and Molecular Medicine, and; ^6^ Division of Clinical Pharmacology, Department of Medicine, University of Cape Town, Cape Town, South Africa;; ^7^ Department of Infectious Diseases, Imperial College, London, United Kingdom;; ^8^ Department of Medical Microbiology and Immunology, Faculty of Medicine, University of Colombo, Colombo, Sri Lanka; and; ^9^ Francis Crick Institute, London, United Kingdom

**Keywords:** Nanopore, *NAT2*, pharmacogenomics, targeted sequencing, tuberculosis

## Abstract

**Rationale:**

Standardized dosing of antitubercular drugs leads to variable plasma drug levels, which are associated with adverse drug reactions, delayed treatment response, and relapse. Mutations in genes affecting drug metabolism explain considerable interindividual pharmacokinetic variability; however, pharmacogenomic assays that predict metabolism of antitubercular drugs have been lacking.

**Objectives:**

We sought to develop a Nanopore sequencing panel and validate its performance in patients with active tuberculosis (TB) to personalize treatment dosing.

**Methods:**

We developed a Nanopore sequencing panel targeting 15 SNPs in five genes affecting the metabolism of antitubercular drugs. For validation, we sequenced DNA samples (*n* = 48) from the 1,000 Genomes Project and compared the variant calling accuracy with that of Illumina genome sequencing. We then sequenced DNA samples from patients with active TB (*n* = 100) from South Africa on a MinION Mk1C and evaluated the relationship between genotypes and pharmacokinetic parameters for isoniazid (INH) and rifampin (RIF).

**Measurements and Main Results:**

The pharmacogenomic panel achieved 100% concordance with Illumina sequencing in variant identification for the samples from the 1,000 Genomes Project. In the clinical cohort, coverage was more than 100× for 1,498 of 1,500 (99.8%) amplicons across the 100 samples. Thirty-three percent, 47%, and 20% of participants were identified as slow, intermediate, and rapid INH acetylators, respectively. INH clearance was 2.2 times higher among intermediate acetylators and 3.8 times higher among rapid acetylators, compared with slow acetylators (*P* < 0.0001). RIF clearance was 17.3% (2.50–29.9) lower in individuals with homozygous *AADAC* rs1803155 G→A substitutions (*P* = 0.0015).

**Conclusions:**

Targeted sequencing can enable the detection of polymorphisms that influence TB drug metabolism on a low-cost, portable instrument to personalize dosing for TB treatment or prevention.

**At a Glance Commentary**

**Scientific Knowledge on the Subject**

Standardized dosing of antitubercular drugs results in variable plasma drug levels, which is associated with adverse drug reactions, poor treatment outcomes, and a risk of relapse. Mutations in genes affecting drug metabolism may explain this pharmacokinetic variability; however, pharmacogenomic assays that predict metabolism of antitubercular drugs have been lacking.

**What This Study Adds to the Field**

We developed a custom, single-tube Nanopore sequencing panel to detect mutations for predicting the metabolism of isoniazid, rifampicin, linezolid, and bedaquiline. Such assays are not currently available in clinical settings to guide drug dosing. We validated our panel on Coriell DNA samples (*n* = 48) and achieved 100% concordance with Illumina whole-genome sequencing data. Next, we validated the predicted metabolism of isoniazid and rifampicin on the basis of genotypes derived from the pharmacogenomic panel in patients with active tuberculosis (TB) (*n* = 100) undergoing treatment and found strong correlation with INH metabolism. Targeted sequencing on an affordable and portable device can facilitate the identification of polymorphisms that impact TB drug metabolism, allowing for personalized dosing in TB treatment or prevention.

Tuberculosis (TB) continues to be a major cause of morbidity and mortality worldwide. Standardized dosing of antitubercular drugs is effective in the treatment and prevention of TB, but it may result in variable plasma drug levels and risk serious drug-related toxicities (1, 2). Studies have shown that a substantial proportion of patients treated for active TB experience at least one type of adverse drug reaction (35–68%), treatment failure (3%), or relapse (6–10%) within 2 years (3–6). Liver enzyme elevations and drug-induced liver injury are the most common adverse effects, affecting up to 30% of patients undergoing standard therapy (7–10). Interindividual drug pharmacokinetic (PK) variation also affects treatment response. In a clinical cohort in South Africa, individuals who had any plasma drug concentration below target levels had a 14-fold increased risk of microbiological failure, death, or relapse (11–13).

A growing body of literature has identified mutations in genes encoding antitubercular drug-metabolizing enzymes that explain substantial PK variation and predict treatment outcomes and risk of adverse events (14, 15). Mutations in N-acetyltransferase 2 (*NAT2*) and cytochrome P450 family 2 subfamily E member 1 (*CYP2E1*) genes are known to affect metabolism and clearance of isoniazid (INH) (16). Polymorphisms in the *NAT2* gene explain up to 88% interindividual PK variability of INH (17, 18). On the basis of mutations in the *NAT2* gene, individuals can be classified into three phenotypes—rapid, intermediate, and slow acetylators. Rapid acetylators typically have the lowest plasma INH concentrations, whereas slow acetylators have the highest (19). The *CYP2E1* gene brings about conversion of acetyl hydrazine to reactive metabolites, which may result in hepatotoxicity (20). Patients with *CYP2E1 Rsa*I polymorphism are significantly less likely to experience hepatotoxicity than those with the wild-type (*1A/*1A) genotype (21, 22). Associations between rifampin (RIF) clearance and mutations in the drug transporter gene solute carrier organic anion transporter family member 1B1 (*SLCO1B1*) and arylacetamide deacetylase (*AADAC*) have also been reported (23, 24). A study in South Africa found that patients with mutation in the *SLCO1B1* gene decreased the area under the plasma drug concentration–time curve (AUC) for RIF (25). Cheli and colleagues found that individuals with mutations in the cytochrome P450 gene cytochrome P450 family 3 subfamily A member 5 *CYP3A5* are at high risk to linezolid (LZD) underexposure (26). However, it should be noted that LZD is primarily metabolized through nonenzymatic processes, with the majority of the drug being eliminated unchanged in the urine (27). The functional role of the CYP3A5 enzyme in LZD metabolism is not known and needs further investigation.

Modification of antitubercular drug doses on the basis of pharmacogenomic (PGx) data can improve PK target attainment, reduce toxicity risk, and improve treatment outcomes. Observational studies have shown that INH dose modifications enabled rapid and slow acetylators to achieve INH AUC targets comparable with those of intermediate acetylators (28, 29). A randomized trial of PGx-guided INH dosing among patients with active TB found that drug-induced liver injury was eliminated in slow acetylators (0% vs. 78% in the standard dosing arm) and early treatment failure was reduced in rapid acetylators (15% vs. 40% in the standard dosing arm) (29, 30).

Despite this evidence, PGx-guided dosing is not widely used in the treatment of TB. A major barrier to its implementation is the lack of scalable assays that can be performed quickly in facilities where TB is treated. At present, PGx testing for *NAT2* and other relevant genes is typically only available in select reference laboratories and is often performed using expensive equipment that is not widely available in clinical laboratories, particularly in low- and middle-income countries where the majority of TB cases occur. In this study, we developed and validated a multiplex targeted sequencing–based panel to detect PGx markers for INH, RIF, LZD, and bedaquiline (BDQ) for use on Nanopore MinION sequencers, which are low-cost instruments that are increasingly accessible worldwide. We further validated our panel in a cohort of patients with active TB who were undergoing treatment, demonstrating the utility to identify PGx determinants of drug metabolism.

Some of the results of these studies have been previously reported in preprint form (30). Content related to this research has been published in abstract form (31).

## Methods

### Selection of PGx Markers

**Figure 1.**
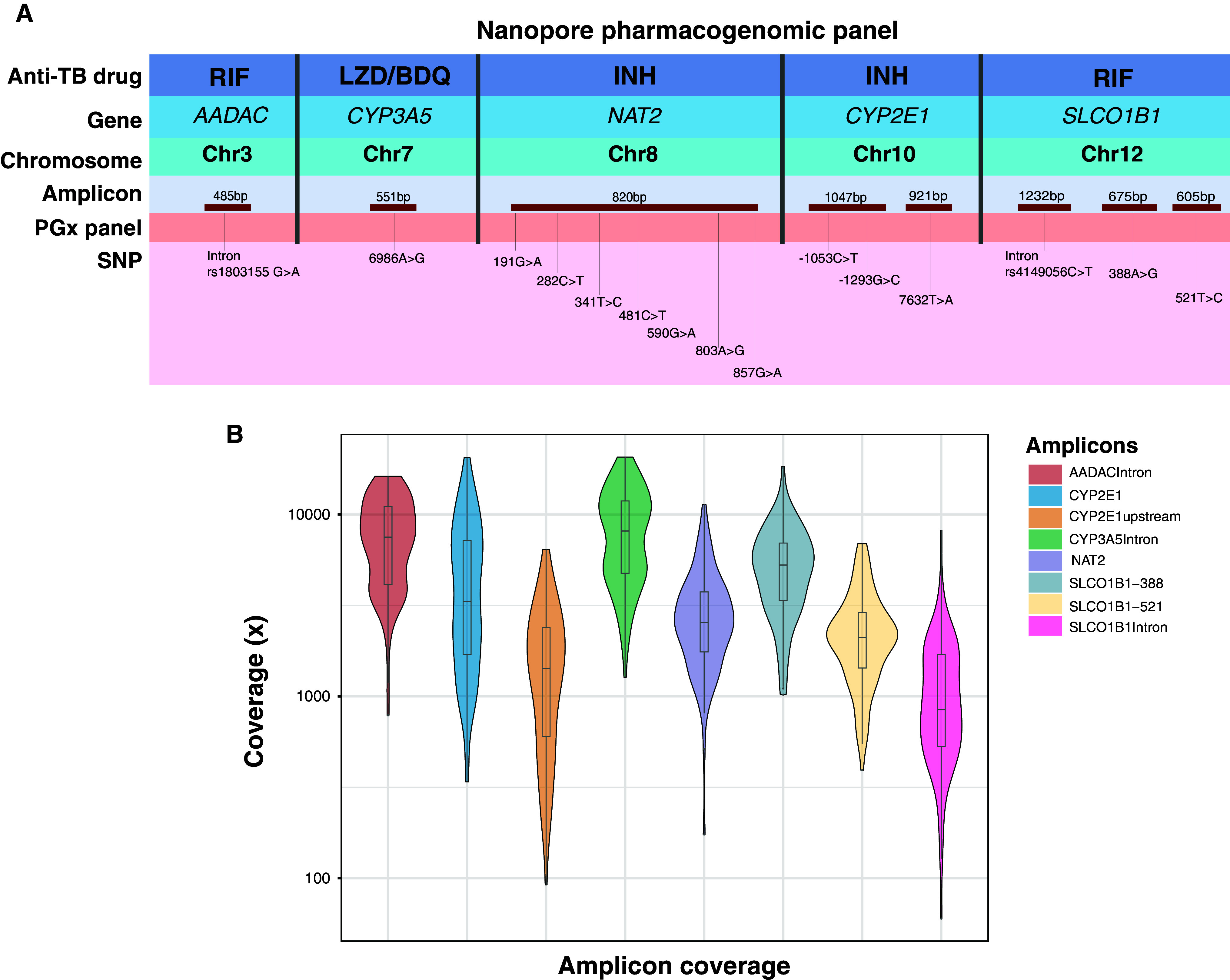
(*A*) Nanopore pharmacogenomic (PGx) panel. The top row indicates the antitubercular drugs for which pharmacogenomic associations were identified from published studies. Genes and their location on the Chr are listed in Rows 2 and 3. On the basis of the position of targeted SNPs, targets were divided into eight amplicons corresponding to five genes (fourth row, red). The amplicons are not scaled to their product length. The last row contains information on the positions in pharmacogenes that were included in the PGx panel. (*B*) Amplicon coverage in PGx panel: sequencing coverage (log_10_ scale) per amplicon in 100 samples from the pharmacokinetic cohort, sequenced on a MinION Mk1C sequencer in a single-tube reaction. BDQ = bedaquiline; chr = chromosome; INH = isoniazid; LZD = linezolid; RIF = rifampin; TB = tuberculosis.

We searched published literature for pharmacogenetic markers of metabolism for drugs that are recommended by the World Health Organization (WHO) for the treatment of TB, including multidrug-resistant or RIF-resistant TB. We selected 15 well-characterized SNPs for which high-quality studies had demonstrated associations with PK parameters, adverse events, or treatment outcomes (18–28). The 15 SNPs, which had PGx associations with INH, RIF, LZD, or BDQ, occurred in five genes: *NAT2*, *CYP2E1*, *SLCO1B1*, *AADAC*, and *CYP3A5*. These variations included 10 SNPs located in exons, three located in introns, and two upstream of an exon (Figure 1A). (For a list of antitubercular drugs with associated pharmacogenes and the impact of mutations on drug metabolism, *see* Table E1 in the online supplement.)

### Primer Design

To perform targeted sequencing, we used a multiplex strategy that relied on anchored primers. We developed an 8-plex panel amplifying regions in five genes. Primers were designed to amplify products between 485 and 1,232 bp using Beacon Designer (*see* Supplemental Methods and Table E2).

### DNA Samples for Panel Development and Validation

For the Nanopore PGx panel development and validation, 48 purified DNA samples from the 1,000 Genomes Project, for which Illumina whole-genome sequencing data were available, were procured from the Coriell Institute for Medical Research (32) (Supplemental Methods).

### Single-Tube Multiplex PCR

A single-tube, 8-plex PCR reaction was performed for each sample using the LongAmp Taq DNA Polymerase (NEB). The final reaction was performed in a 50-μl volume containing 1× LongAmp Taq Reaction Buffer (NEB), 2.5 M Betaine (Sigma), and 5 U LongAmp Taq DNA Polymerase. Primer efficiency was first evaluated on a single-plex reaction with the subsequent addition of primer sets to evaluate the nonspecific binding and inhibition of each primer set by the others. The final PCR conditions were optimized to 2 min of DNA denaturation at 94°C followed by 30 cycles of amplification as follows: 30 s at 94°C for denaturing, 60 s at 60°C for primer annealing, 1.5 min at 65°C for extension, followed by 10 min at 65°C for a final extension. The PCR product was purified with the PureLink PCR Purification Kit (Thermo Fisher Scientific). The purified PCR product was eluted with 50 μl nuclease-free water and quantified with Qubit to evaluate reaction yield.

### MinION Library Preparation and Sequencing

For panel development and validation, we sequenced a total of 60 Coriell DNA samples (one Coriell DNA sample at 12 dilutions and 48 additional Coriell DNA samples with different genotypes). The library was prepared using the SQK-LSK110 Ligation Sequencing Kit (Oxford Nanopore Technologies). Samples were barcoded using a Nanopore PCR barcoding expansion (EXP-PBC096 PCR Barcoding Expansion) followed by DNA repair and end-prep using NEBNext FFPE DNA Repair Mix and NEBNext Ultra II End repair/dA-tailing Module reagents in accordance with the manufacturer’s instructions. Adaptor ligation was performed using Adapter Mix F (AMX-F) and Quick T4 Ligase. The samples were sequenced on a MinION Mk1C sequencer (*see* Supplemental Methods and Table E3).

### Sequencing Data Analysis

Demultiplexing and real-time basecalling were performed on in-built MinKNOW software (Release 22.08.4) using the onboard basecalling software Guppy (Version 3). The run was set on a high accuracy basecalling (cutoff, >9). Mapping was conducted by aligning reads to a multi-FASTA file containing the concatenated sequences of the genome regions included in the panel. Reference FASTA genome was uploaded on the Epi2ME (Version 4.1.3.) cloud and fastq files with passed reads well aligned to the custom genome to generate bam files. To call variants first, the reads were mapped to the reference sequences of target genes included in the gene panel using Minimap2 (Version 2.26) with default parameters. Aligned reads with a mapping quality score under 60 (MAPQ60) were discarded. Variant calling was performed with Calir3 using default ONT settings. Variants identified were phased using WhatsHap (Version 1.7).

### Samples for Clinical Validation

#### Study Cohort and Ethical Approval

Patients with RIF-susceptible pulmonary TB, as confirmed with the GeneXpert MTB/RIF assay, were recruited at the Ubuntu HIV/TB Clinic, Site B, Khayelitsha, South Africa (University of Cape Town Faculty of Health Sciences Human Research Ethics Committee approval 568/2012) as a part of a larger study. Whole-blood samples (*n* = 100) from a subcohort of this study who were invited to participate in a nested PK study between July 2013 and April 2014 were used for PGx validation (33). Ethical approval was obtained from the University of Cape Town Human Research Ethics Committee for the storage and future PDx testing of DNA from whole-blood samples at the time of sample collection (HREC Reference No. 568/2012). All patients provided written consent before participation. Detailed sociodemographic data, past TB treatment history, and comorbidity data were collected. Weight band–based dosing was used in line with WHO guidelines (34) (Table 1).

**Table 1.** Clinical Characteristics of TB PK Cohort

Clinical Characteristic	PK Cohort (*N* = 100)
Female sex, *n*	43
Age in years, median (IQR)	33 (29–40)
Smear grade at baseline, *n*	
3+	24
2+	22
1+	20
Scanty or negative, *n*	34
Baseline time to culture positivity, d, median (IQR)	10 (7–14)
Extensive radiological disease at baseline, *n*	71
Cavities at baseline, *n*	52
Smoking history, n*	
Current	24
Previous	27
Never	49
Alcohol consumption, *n*^†^	37
Retreatment, *n*^‡^	39
Type 2 diabetes mellitus, *n*^§^	4
BMI at PK study, kg/m^2^, median (IQR)	21.5 (20–23)
Albumin concentration at PK study, g/L, median (IQR)	38 (34–40)
Dose, mg/kg, median (IQR)	
Rifampin	10 (7–11.5)
Isoniazid	5 (3.5–6)
Participants reporting side effects of TB treatment, *n* (%)	35 (35)

*Definition of abbreviations*: BMI = body mass index; IQR = interquartile range; PK = pharmacokinetic; TB = tuberculosis.

Numerical values reflect number of participants with the characteristic unless otherwise specified.

* Smoking was ascertained as current, previous, or never.

^†^ Alcohol use (days per week) was ascertained as none, less than one drink per day, one to five drinks per day, six to seven drinks per day/occasional alcohol use (drinks per day) (not applicable, one to two drinks per day, three to five drinks per day, or more than six drinks per day).

^‡^ Previous treatment for active TB disease, episode year, months of treatment received, outcome (cure, treatment completion, default, failure, or unknown).

^§^ Diabetes mellitus was defined as HbA1C more than 7%, fasting plasma glucose of 7.0 mmol/L or more, or oral glucose tolerance test result more than 11.1 mmol/L, which was performed in a subcohort that was also being corecruited to an ongoing diabetes-TB comorbidity study; additionally, it was recorded as present if the patient self-reported as having diabetes and was currently taking diabetes medications.

#### PK Data

A description of the sampling, therapeutic drug monitoring, and PK analysis for this cohort has previously published (35). Briefly, PK sampling was performed for RIF and INH 7 to 8 weeks after the initiation of antitubercular therapy. Blood samples were obtained immediately before (predose) and 1, 2, 3, 4, 6, and 8 hours after drug ingestion. They were immediately placed on ice, and plasma was separated by centrifugation within 30 minutes before storage at −80°C until analysis. The storage tubes containing the plasma samples were transferred on dry ice to the Clinical Pharmacology Laboratory at the University of Cape Town, where drug concentrations were determined using validated liquid chromatography tandem mass spectrometry methods (35).

Whole-blood samples from 100 TB-positive patients for which PK data were available were collected in citrate tubes and stored in −80°C until used. DNA was extracted from 100 μl whole-blood samples using the DNeasy Blood and Tissue Kit (QIAGEN) and eluted in 50 μl nuclease-free water. Approximately 50 ng purified DNA was used for targeted sequencing using a custom Nanopore PGx panel as described earlier.

#### Haplotype Labeling

For INH PGx analysis, phased *NAT2* haplotypes for a PK cohort derived from Nanopore sequencing were labeled on the basis of seven canonical SNPs following an international consensus nomenclature to interpret acetylator phenotype (36). The *CYP2E* allele nomenclature was quoted on the basis of the Human Cytochrome P450 Allele Nomenclature Committee tables (37).

### Statistical Analyses

We compared demographic and clinical characteristics by acetylator group using Kruskal-Wallis and chi-square tests. Previously developed population PK models were used to test the effect of the polymorphisms or *NAT2* haplotypes (acetylator type) listed in Table 2 on clearance and bioavailability categorically within the models. These models included body size on all disposition parameters (allometric scaling), HIV on INH clearance, and both HIV and lopinavir/ritonavir use on rifampicin clearance. The models were developed using nonlinear mixed-effects modeling in Monolix (Version 2023R1; Lixoft SAS). Goodness-of-fit and statistical significance were assessed using likelihood-ratio tests based on the −2 log-likelihood (−2LL) provided by the software and assumed to follow a chi-square distribution. We performed stepwise covariate selection using a drop in −2LL (Δ−2LL) of more than 3.84 as a cutoff for inclusion in forward inclusion (corresponding with a *P* < 0.05) and an increase of more than 6.64 as a cutoff for retention in backward elimination (*P* < 0.01). Model-based individual PK parameters were derived from the final model. Pre- and postprocessing of data was conducted in R (Version 4.3.1). Final population parameter estimates for rifampicin and isoniazid are provided in the online supplement tables E4 and E5 respectively.

**Table 2.** Variant Calling Summary of 15 PGx Panel Markers for 100 Clinical Cohort Samples Analyzed on a Nanopore MinION Sequencer

Pharmacogene	SNP Identifier	SNP	WT (*%*)	MUT (*%*)	HET (*%*)	Genotype Quality, Median	Read Depth, Median
*NAT2*	rs1801279	191G→A	87	2	11	20.0	4,801.0
*NAT2*	rs1041983	282C→T	41	17	42	22.0	5,180.0
*NAT2*	rs1801280	341T→C	57	4	39	23.0	4,775.0
*NAT2*	rs1799929	481C→T	65	3	32	21	4,976.0
*NAT2*	rs1799930	590G→A	54	4	42	21.0	5,339.0
*NAT2*	rs1208	803A→G	29	21	50	20.0	4,715.0
*NAT2*	rs1799931	857G→A	99	0	1	23.0	6,457.0
*CYP2E1*	rs3813867	−1293G→C	93	0	7	21.0	5,377.0
*CYP2E1*	rs2031920	−1053C→T	100	0	0	NA	NA
*CYP2E1*	rs6413432	7632T→A	90	0	10	20.0	8,377.0
*SLCO1B1*	rs4149032	C→T	0	70	30	24.0	1,721.0
*SLCO1B1*	rs4149056	521T→C	100	0	0	NA	NA
*SLCO1B1*	rs2306283	388A>G	2	66	32	24.0	4,979.0
*AADAC*	rs1803155	G>A/T	2	65	33	23.0	8,047.0
*CYP3A5*	rs776746	6986A>G	75	1	24	21.0	8,053.0

*Definition of abbreviations*: HET = heterozygous; MUT = homozygous mutant; NA = not applicable; PGx = pharmacogenomic; WT = wild-type.

## Results

### PGx Panel Performance and Coverage per Amplicon

To evaluate the panel’s sensitivity and accuracy, we first performed targeted Nanopore sequencing on six samples in duplicates that were obtained by diluting a Coriell DNA sample with known genotype. At all dilutions, we obtained median coverage above the minimum cutoff (>50×). Coverage from 500 ng to 50 ng was 850×, 847×, 972×, 1,005×, 1,066×, and 1,076×, respectively. The median quality score of diluted samples was 12.8 (SD = 0.1), and the median yield for passed reads was 15 Mb (SD = 2.4) (*see* Figure E1). We then validated the PGx panel on 48 purified DNA samples from Coriell Institute. The majority of the Coriell samples selected for panel validation were from sub-Saharan Africa (54.1%) and the Americas (25%) and were 41.3% male. We achieved complete coverage of the targeted regions by aligning eight PCR amplicons in the PGx panel. All amplicons were sequenced with coverage depth above the minimum cutoff. The median sequencing depth across eight amplicons in Coriell samples was ∼2,281×, with 99.7% of the amplicons above 100× and 90.2% above 500×. Among the eight amplicons, CYP3A5Intron had the highest coverage (median = 6,934×; IQR = 2,522.0–9,534.7), and SLCO1B1Intron had the lowest coverage (median = 779; IQR= 429.5–1,221.5). We observed 100% concordance between variants identified in the Nanopore PGx panel and the reference Illumina whole-genome sequencing.

### Clinical Validation

For clinical validation, we performed targeted Nanopore sequencing on DNA extracted from whole-blood samples from active TB patients enrolled in the INH and RIF PK cohort (Table 1). A majority of the participants were of Xhosa ethnicity (98/100; 98%), and 65% of the participants were living with HIV. The median age was 33 years (range = 29–40), and 43% were women. The median quality score of the samples was 13.8 (IQR = 13.5–14.0). We obtained full coverage of the targeted regions for every sample, with a coverage depth that exceeded the minimum cutoff (>50×) for all amplicons. The median sequencing depth across eight amplicons in the PK cohort was ∼2,963 × (IQR = 1,512–6,156), with 99.8% amplicons above 100× and 93.6% above 500× (Figure 1B).

### INH and RIF PGx Associations

**Figure 2.**
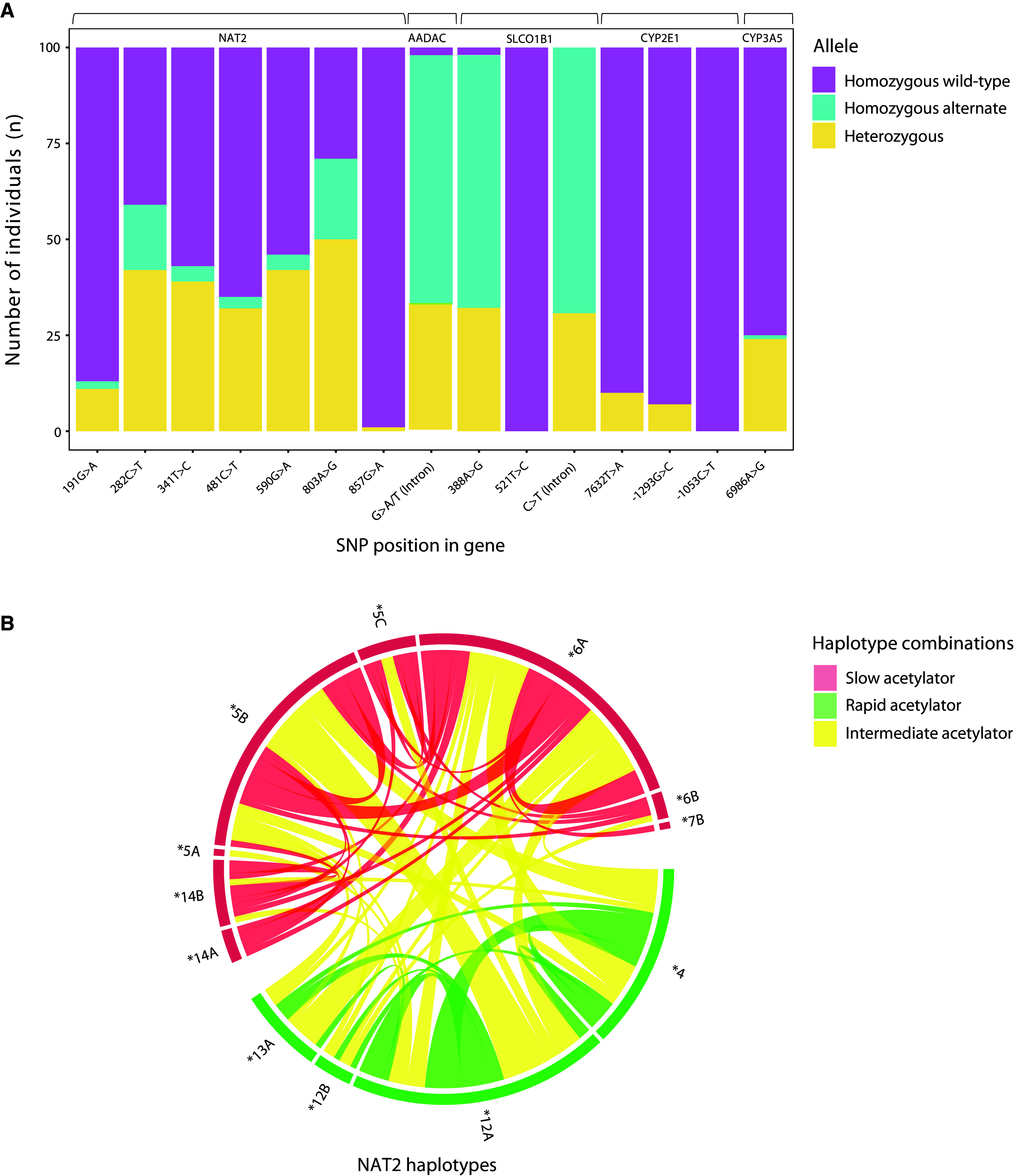
(*A*) Distribution of homozygous wild-type (purple), homozygous alternate (blue), and heterozygous (yellow) alleles at 15 polymorphic sites in patients with active tuberculosis (*n* = 100) from a pharmacokinetic cohort sequenced on a MinION sequencer. (*B*) *NAT2* haplotypes indicated in red are slow acetylator types, and those indicated in green are rapid acetylator haplotypes. Connections in red indicate two slow acetylator haplotypes, those in green indicate two rapid haplotypes, and those in yellow indicate one rapid and one slow haplotype (intermediate acetylation).

We obtained a read depth above 100× for variant alleles at all positions. A total of 253 homozygous mutant and 353 heterozygous alleles were detected in 100 samples at 15 genomic positions. The frequency of homozygous wild-type, homozygous alternate, and heterozygous variant alleles is shown in Figure 2A and Table 2.

**Figure 3.**
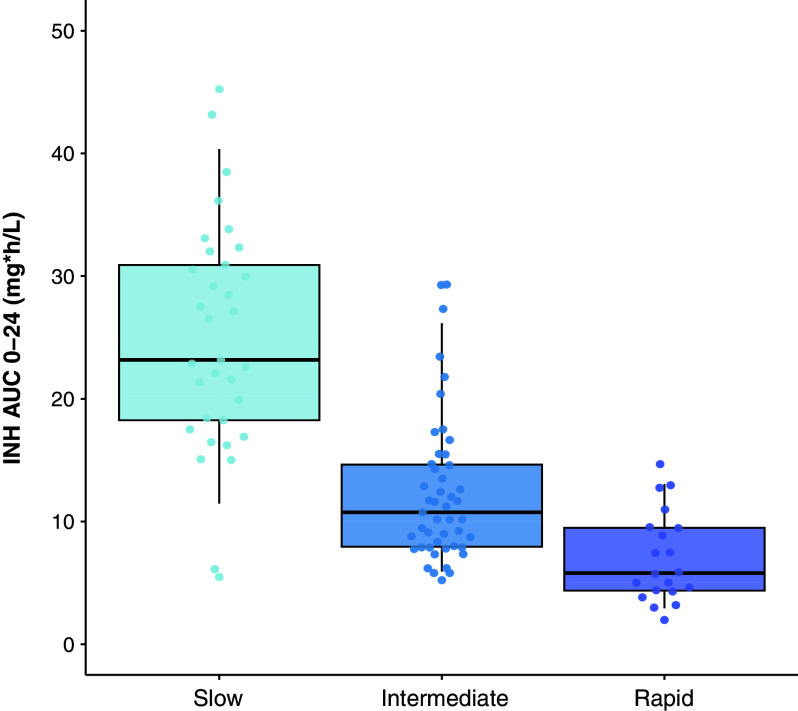
The area under the plasma drug concentration–time curve (AUC) for 0–24 hours in INH. Predicted slow acetylators (light blue), intermediate acetylators (blue), and rapid acetylators (purple). AUC was lowest for rapid acetylators, moderate for intermediate acetylators, and highest in slow acetylators. INH = isoniazid.

On the basis of the international consensus nomenclature, participants were classified as slow (33/100; 33%), intermediate (47/100; 47%) and rapid (20/100; 20%) acetylators. Demographic and clinical characteristics did not differ by acetylator type (Table 3). The *NAT2* haplotype distribution for 100 PK samples is provided in Figure 2B. *NAT2* acetylator status had a significant effect on INH clearance with typical clearance 2.3 times higher among intermediate acetylators and 3.8 times higher among rapid acetylators compared with slow acetylators (Δ−2LL = 105.5, *P* < 0.0001) (*see* Figure E2). The AUC for 0–24 hours was lowest for rapid acetylators (median, 5.8 mg * h/L; IQR, 4.3–9.5), moderate for intermediate acetylators (median, 10.6 mg * h/L; IQR, 7.9–14.6) and highest in slow acetylators (median, 26.1 mg ⋅ h/L; IQR, 18.1–31.2) (Figure 3 and Table 3). After the inclusion of acetylator status, 7632T→A (rs6413432) had a significant effect on INH bioavailability. Participants who were heterozygotes (*n* = 10) had a 23% (2.2–50) higher bioavailability than wild-type patients (*P* = 0.0008). However, we did not retain this effect in the final model because of high parameter uncertainty that was due to a low number of patients in the heterozygous group. In addition, the effect of HIV, which was included in the previously published model, was now no longer significant in the backward elimination after including the effect of SNPs (Δ−2LL = 2.31, *P* = 0.130), so it is no longer present in the final model. Of note, individuals who were slow acetylators were more likely to report side effects than intermediate or fast acetylators (52% vs. 27%, *P* = 0.027).

**Table 3.** Demographic and Clinical Characteristics, and INH PK Parameter Estimates, by *NAT2* Acetylator Status

Demographic	Slow (*n* = 33)	Intermediate (*n* = 47)	Rapid (*n* = 20)	*P* Value*
Age, yr, median (IQR	33.4 (28.9–39.0)	32.4 (30.3–40.6)	29.5 (27.1–41.6)	0.44
Sex, female, *n* (%)	16 (48)	19 (40)	8 (40)	0.739
Albumin, median (IQR)	39 (36.0–42.0)	39.0 (34.0–43.0)	39.5 (36.3–76.3)	0.785
Days on TB treatment, median (IQR)	56.0 (53.0–57.0)	56.0 (52.0–60.5)	54.5 (48.8–58.0)	0.455
HIV infected, *n* (%)	8 (24)	19 (40)	8 (40)	0.286
BMI, kg/m^2^, median (IQR)	22 (19.3–23.0)	22.0 (19.3–23.0)	21.5 (19.8–23.0)	0.532
INH PK, median (IQR)				
AUC, mg * h/L	26.0 (17.9–30.9)	10.6 (7.9–14.6)	5.8 (4.3–9.5)	^†^
C_max_, mg/L	4.5 (3.3–5.6)	3.2 (2.6–4.6)	2.4 (1.6–3.5)	^†^

*Definition of abbreviations*: AUC = area under the plasma drug concentration–time curve; BMI = body mass index; C_max_ = maximum serum concentration of a drug; INH = isoniazid; IQR = interquartile range; PK = pharmacokinetic; TB = tuberculosis.

* We used Kruskal–Wallis and chi-square tests to compare demographic and clinical characteristics by group.

^†^ The values reported are model-derived individual PK parameters and are shown only for illustrative purposes. A statistical difference between the groups was established as an effect on clearance in the population PK model (*P* < 0.0001).

We also evaluated whether polymorphisms in *CYP2E1*, part of the downstream INH metabolism pathway, could explain PK variability or were associated with adverse events. Eighty-seven participants were *1A/1A (wild-type) genotype, 7 were *5B/*5A, and 6 were *1A/*6. We did not observe any significant associations (*P* = 0.28) between *CYP2E1* haplotypes and reported side effects.

**Figure 4.**
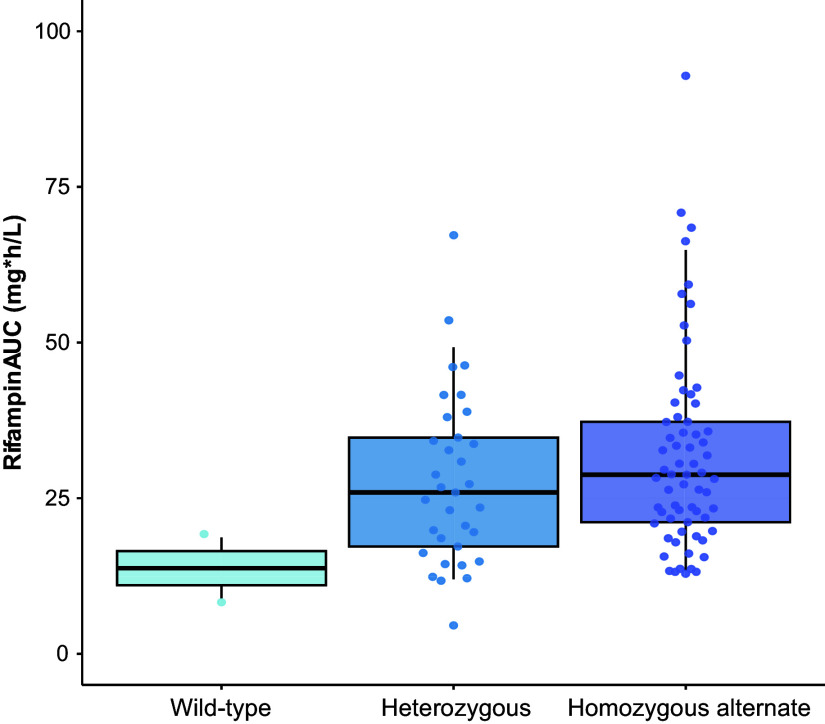
The area under the plasma drug concentration–time curve (AUC) for 0–24 hours in rifampin. Homozygous alternate (purple) for *AADAC* rs1803155 G→A substitutions, heterozygous (blue), and homozygous wild-type (light blue) alleles.

For RIF PGx analysis, we analyzed three SNP sites in *SLCO1B1* (rs4149032 C→T, 388A→G, and 521T→C) and one in *AADAC* (rs1803155). At the rs4149032 position, 70/100 patients were homozygous mutant and 30/100 were heterozygous alleles. We identified 2/100 wild-type, 66/100 homozygous alternate, and 32/100 heterozygous alleles at the 388A→G position. All samples were detected as wild-type at the 521T→C position in the *SLCO1B1* gene. We did not observe any significant associations between *SLCO1B1* mutations and RIF bioavailability. RIF clearance was 16.5% (1.30–29.3) lower in individuals who were homozygous alternate for *AADAC* rs1803155 G→A substitutions (*P* = 0.0015; Figure 4 and *see* Figure E3).

## Discussion

Although TB is treatable and preventable, a substantial proportion of patients experience drug-associated toxicities, treatment failure, and relapse under standardized dosing. For preventive therapy, adverse drug events, which are associated with drug metabolism, are a strong predictor of noncompletion (38). PGx-guided dosing has the potential to reduce the risk of these poor outcomes, with observational studies and a randomized trial demonstrating strong premise for feasibility and effectiveness (39). However, a major obstacle to using PGx-guided dosing is the lack of access to PGx assays in clinical settings where TB is common. To address this gap, we developed a single-tube, targeted-sequencing panel on the Oxford Nanopore MinION platform to detect mutations associated with the metabolism of INH, RIF, LZD, and BDQ for which PGx associations were previously reported. We achieved high coverage and read depth for all targets in the panel and found that variant identification was 100% concordant in well-characterized reference genomes. As proof of principle, we performed the assays on samples from an active TB clinical cohort in Cape Town, confirming that *NAT2* acetylator types strongly predicted INH clearance in this population.

Currently available methods used for the detection of mutations in pharmacogenes largely rely on qPCR, restriction fragment length polymorphisms, SNP array platforms, single-gene Sanger sequencing, or larger scale (exome or whole-genome) sequencing (40). Although qPCR methods are rapid and easier to perform, they target only a limited number of mutations and provide unphased data and *in silico* haplotype predictions. One consequence of this is that polymorphisms that important in some populations are sometimes neglected. For example, the G191A (R64Q) SNP is common to the NAT2*14 allele cluster, which is frequent in African and African-American individuals but is rarely observed in other populations (41), leading it to be left out of the popular *NAT2* phasing tool *nat2pred* (42). One study found no correlation between the *NAT2* genotype and INH metabolism in individuals of Zulu descent in South Africa; however, the study excluded the G191A SNP, leading to a population-specific prediction bias (43). SNP array platforms and whole-exome or whole-genome sequencing provide data covering more genes and relevant SNPs, but typically require expensive laboratory equipment and are not widely available in clinical facilities in resource-constrained settings.

We previously developed a qPCR-based PGx assay on the GeneXpert platform to detect polymorphisms in the *NAT2* gene to guide INH dosing (44). This assay predicted INH metabolism with high accuracy on the basis of five canonical SNPs; however, there are constraints to including further targets for a single-tube cartridge-based assay. As TB treatment requires at least three drugs, and many drugs have several relevant pharmacogenes and multiple important SNPs per gene, an optimal panel will require multiple targets. We identified 15 SNPs in five genes for which there are compelling PGx data for important antitubercular drugs. Our search identified many other SNPs for which data were sparse or conflicting; as further studies confirm or reject associations between these polymorphisms, our assay could be easily expanded to include other targets.

In the present study, which covered all seven canonical SNPs in *NAT2*, we found that *NAT2* haplotypes were strongly predictive of isoniazid clearance and AUC; clearance was nearly four times higher, and AUC was four times lower, in rapid acetylators compared with slow acetylators. Prior studies have demonstrated that increasing isoniazid dosing among rapid acetylators (to 7.5–10 mg/kg) and decreasing it among slow acetylators (to 2.5 mg/kg) can achieve PK targets and reduce adverse events (29). Given the diversity of *NAT2* acetylator types in this population and globally, testing combined with isoniazid dose modification could confer substantial clinical benefits (29). We found a modest effect of rs1803155 G→A substitution in *AADAC*; homozygous individuals had 17.3% lower clearance than heterozygous and wild-type alleles (*P* = 0.0015). In our study, all participants were heterozygous or homozygous alternate for the rs4149032 polymorphism, so we lacked a reference group of wild-type individuals for comparison. The high frequency of rs4149032 polymorphisms in this population and the data showing that a low RIF AUC is predictive of poor outcomes add to the growing evidence that higher doses of RIF may be needed (45, 46).

We included pharmacogenetic targets associated with LZD and BDQ toxicity, although these are not first-line TB drugs. LZD and BDQ are now both included in the primary WHO-recommended regimen for treatment of multidrug-resistant or RIF-resistant TB (47, 48). Additionally, a recent trial demonstrated that an 8-week course including LZD and BDQ for RIF-susceptible TB was noninferior to standard 6-month therapy, opening the door to potential ultrashort course regimens (48). Both drugs are associated with serious adverse events. Peripheral neuropathy and myelosuppression are common with prolonged courses of LZD and can be treatment limiting. Prolongation of the QT interval (the time from the start of the Q wave to the end of the T wave), leading to serious arrythmias, can be seen with BDQ, particularly when used in combination with other drugs that prolong the QT interval, including moxifloxacin. The CYP3A5 *1 haplotype was associated with a nearly sixfold risk of LZD underexposure, compared with *3/*3, and the *3 haplotype was associated with slower clearance of BDQ, including 30% lower clearance for homozygous individuals (*3/*3) (26–28). Although we analytically validated our assay to correctly identify these polymorphisms, our clinical cohort did not include individuals receiving these drugs, and further studies are needed to confirm the importance of these variants in diverse populations.

Amplicon-based approaches coupled with MinION sequencing offers several advantages over conventional methods. MinION supports real-time base calling that allows users to stop or pause the run when the output is enough for the analysis. This is highly advantageous when used in clinical settings where quick results are needed to decide treatment, improve prognosis, and guide clinical management. A recent study used a custom variant-prioritization approach with Nanopore sequencing to rapidly diagnose various disorders in critically ill patients (49). In the present study, we performed 24-hour and 48-hour runs, resulting in coverage that was several-fold higher than the required cutoff value. On the basis of these findings, the run time of the PGx assay can be cut down to a few hours. Targeted Nanopore sequencing has been previously used to detect drug-resistant strains in *Mycobacterium tuberculosis* from sputum (50) and the point-of-care diagnosis of viral and bacterial infections (51, 52). Another advantage of using the Nanopore sequencing approach is the availability of a smaller and cheaper Flongle flowcells (∼$90), which produce up to 2.8 Gb of output. We found that we could sequence 50 samples on each Flongle run with sufficient coverage. Therapeutic drug monitoring (TDM), where available, can be a useful way to optimize dosing at the individual level. However, TDM is performed after starting therapy, and results are often not available for the first few weeks of therapy, when optimal dosing is most critical for reducing bacillary burden. In such settings, PGx testing can inform initial dosing, and TDM can be used to further refine and optimize drug levels. However, over 95% of TB cases globally occur in low- and middle-income countries, where TDM is not widely available; in such settings, an accessible PGx assay could potentially improve dosing.

Despite these advantages, one of the major challenges in using a sequencing-based diagnostic assay are the DNA extraction and library preparation, which require hands-on time, equipment, and expertise. Automated DNA extraction and library preparation tools are under development and could potentially increase accessibility of sequencing-based assays. Additionally, unlike PCR-based diagnostics, the data processing and analysis workflow is not as straightforward, and automated analytic pipelines are needed to facilitate accessibility. As the WHO now recommends Nanopore and Illumina sequencing–based assays for *M. tuberculosis* drug-resistance determinants, the relevant equipment and expertise may be increasingly available in public health laboratories in TB-endemic settings. Another advantage of amplicon sequencing over PCR-based SNP assays is that, because targeted sequencing provides information on the entire gene sequence or the targeted amplicons, novel mutations can be identified in these targets, offering valuable insights into the evolution of these genes in different populations. Our assay requires single-tube PCR amplification and library preparation before sequencing on Nanopore; instruments to automate these processes are becoming available, which will be important for amplicon sequencing assays to be implemented in clinical laboratories.

The findings of this study are subject to several limitations. To increase the efficiency of the multiplex assay, we split the target genes into two or more amplicons covering a region of the gene instead of the full length. Because of this, we may have missed novel mutations in those regions. Although, we achieved high coverage for all amplicons, there was moderate variability in coverage across the amplicons. Furthermore, although we developed the custom panel for four antitubercular drugs, PK data were available only for INH and RIF to predict PGx associations, and there was a lack of genetic diversity at some sites that had been previously identified as important for metabolism of these drugs. Further studies are needed in diverse populations to assess the impact of *CYP3A5* mutations in LZD and BDQ metabolism. Liver enzyme data were not available for assessment of the effect of *CYP2E1* mutations on drug-induced liver injury, which has been previously reported in several studies (22).

Amid growing evidence that we can identify individuals at greatest risk of antitubercular drug toxicities and poor treatment outcomes by screening for common genetic variants, there is a need for assays that can be performed near the point of clinical care in settings where TB is common. We developed and validated a Nanopore amplicon sequencing panel to detect PGx markers for key first- and second-line antitubercular drugs. This panel can be further expanded as additional pharmacogenetic markers of TB medications are identified and validated. The one-time purchase cost of the Nanopore MinION devices is around $1,000, and these sequencers are increasingly available in public health laboratories in low- and middle-income countries. The movement to optimize TB treatment for each patient will require tools such as this that are scalable for use in settings where TB burden is greatest.

## References

[1] Merle CS Fielding K Sow OB Gninafon M Lo MB Mthiyane T *et al.* OFLOTUB/Gatifloxacin for Tuberculosis Project A four-month gatifloxacin-containing regimen for treating tuberculosis *N Engl J Med* 2014 371 1588 1598 25337748 10.1056/NEJMoa1315817

[2] Dorman SE Nahid P Kurbatova EV Goldberg SV Bozeman L Burman WJ *et al.* AIDS Clinical Trials Group and the Tuberculosis Trials Consortium High-dose rifapentine with or without moxifloxacin for shortening treatment of pulmonary tuberculosis: study protocol for TBTC study 31/ACTG A5349 phase 3 clinical trial *Contemp Clin Trials* 2020 90 105938 31981713 10.1016/j.cct.2020.105938 PMC7307310

[3] Fei CM Zainal H Ali IAH Evaluation of adverse reactions induced by anti-tuberculosis drugs in Hospital Pulau Pinang *Malays J Med Sci* 2018 25 103 114 10.21315/mjms2018.25.5.10 PMC6419878 30914867

[4] Forget EJ Menzies D Adverse reactions to first-line antituberculosis drugs *Expert Opin Drug Saf* 2006 5 231 249 16503745 10.1517/14740338.5.2.231

[5] Choi H Park HA Hyun IG Kim JH Hwang YI Jang SH *et al.* Incidence and outcomes of adverse drug reactions to first-line anti-tuberculosis drugs and their effects on the quality of life: a multicenter prospective cohort study *Pharmacoepidemiol Drug Saf* 2022 31 1153 1163 35909258 10.1002/pds.5513

[6] Luzze H Johnson DF Dickman K Mayanja-Kizza H Okwera A Eisenach K *et al.* Tuberculosis Research Unit Relapse more common than reinfection in recurrent tuberculosis 1-2 years post treatment in urban Uganda *Int J Tuberc Lung Dis* 2013 17 361 367 23407224 10.5588/ijtld.11.0692 PMC6623981

[7] Mitchell I Wendon J Fitt S Williams R Anti-tuberculous therapy and acute liver failure *Lancet* 1995 345 555 556 7786350 10.1016/s0140-6736(95)90468-9

[8] Sant’Anna FM Araújo-Pereira M Schmaltz CAS Arriaga MB de Oliveira RVC Andrade BB *et al.* Adverse drug reactions related to treatment of drug-susceptible tuberculosis in Brazil: a prospective cohort study *Front Trop Dis* 2022 2 748310

[9] Yee D Valiquette C Pelletier M Parisien I Rocher I Menzies D Incidence of serious side effects from first-line antituberculosis drugs among patients treated for active tuberculosis *Am J Respir Crit Care Med* 2003 167 1472 1477 12569078 10.1164/rccm.200206-626OC

[10] Shu CC Lee CH Lee MC Wang JY Yu CJ Lee LN Hepatotoxicity due to first-line anti-tuberculosis drugs: a five-year experience in a Taiwan medical centre *Int J Tuberc Lung Dis* 2013 17 934 939 23743313 10.5588/ijtld.12.0782

[11] Gumbo T Louie A Liu W Brown D Ambrose PG Bhavnani SM *et al.* Isoniazid bactericidal activity and resistance emergence: integrating pharmacodynamics and pharmacogenomics to predict efficacy in different ethnic populations *Antimicrob Agents Chemother* 2007 51 2329 2336 17438043 10.1128/AAC.00185-07 PMC1913269

[12] Pasipanodya JG McIlleron H Burger A Wash PA Smith P Gumbo T Serum drug concentrations predictive of pulmonary tuberculosis outcomes *J Infect Dis* 2013 208 1464 1473 23901086 10.1093/infdis/jit352 PMC3789573

[13] Pasipanodya JG Srivastava S Gumbo T Meta-analysis of clinical studies supports the pharmacokinetic variability hypothesis for acquired drug resistance and failure of antituberculosis therapy *Clin Infect Dis* 2012 55 169 177 22467670 10.1093/cid/cis353 PMC3491771

[14] Sahu RK Singh K Subodh S Adverse drug reactions to anti-TB drugs: pharmacogenomics perspective for identification of host genetic markers *Curr Drug Metab* 2015 16 538 552 26264201 10.2174/1389200216666150812123725

[15] Metushi I Uetrecht J Phillips E Mechanism of isoniazid-induced hepatotoxicity: then and now *Br J Clin Pharmacol* 2016 81 1030 1036 26773235 10.1111/bcp.12885 PMC4876174

[16] Kinzig-Schippers M Tomalik-Scharte D Jetter A Scheidel B Jakob V Rodamer M *et al.* Should we use N-acetyltransferase type 2 genotyping to personalize isoniazid doses? *Antimicrob Agents Chemother* 2005 49 1733 1738 15855489 10.1128/AAC.49.5.1733-1738.2005 PMC1087660

[17] Du H Chen X Fang Y Yan O Xu H Li L *et al.* Slow N-acetyltransferase 2 genotype contributes to anti-tuberculosis drug-induced hepatotoxicity: a meta-analysis *Mol Biol Rep* 2013 40 3591 3596 23277397 10.1007/s11033-012-2433-y

[18] Zabost A Brzezińska S Kozińska M Błachnio M Jagodziński J Zwolska Z *et al.* Correlation of N-acetyltransferase 2 genotype with isoniazid acetylation in Polish tuberculosis patients *BioMed Res Int* 2013 2013 853602 24383060 10.1155/2013/853602 PMC3871508

[19] Nelson SD Mitchell JR Timbrell JA Snodgrass WR Corcoran GB III Isoniazid and iproniazid: activation of metabolites to toxic intermediates in man and rat *Science* 1976 193 901 903 7838 10.1126/science.7838

[20] Stephens EA Taylor JA Kaplan N Yang CH Hsieh LL Lucier GW *et al.* Ethnic variation in the CYP2E1 gene: polymorphism analysis of 695 African-Americans, European-Americans and Taiwanese *Pharmacogenetics* 1994 4 185 192 7987402 10.1097/00008571-199408000-00002

[21] Huang YS Chern HD Su WJ Wu JC Chang SC Chiang CH *et al.* Cytochrome P450 2E1 genotype and the susceptibility to antituberculosis drug-induced hepatitis *Hepatology* 2003 37 924 930 12668988 10.1053/jhep.2003.50144

[22] Richardson M Kirkham J Dwan K Sloan DJ Davies G Jorgensen AL CYP genetic variants and toxicity related to anti-tubercular agents: a systematic review and meta-analysis *Syst Rev* 2018 7 204 30458875 10.1186/s13643-018-0861-z PMC6247669

[23] Chigutsa E Visser ME Swart EC Denti P Pushpakom S Egan D *et al.* The SLCO1B1 rs4149032 polymorphism is highly prevalent in South Africans and is associated with reduced rifampin concentrations: dosing implications *Antimicrob Agents Chemother* 2011 55 4122 4127 21709081 10.1128/AAC.01833-10 PMC3165308

[24] Weiner M Gelfond J Johnson-Pais TL Engle M Johnson JL Whitworth WC *et al.* Pharmacokinetics/Pharmacodynamics Group of Tuberculosis Trials Consortium Decreased plasma rifapentine concentrations associated with AADAC single nucleotide polymorphism in adults with tuberculosis *J Antimicrob Chemother* 2021 76 582 586 33374006 10.1093/jac/dkaa490 PMC7879139

[25] Weiner M Peloquin C Burman W Luo CC Engle M Prihoda TJ *et al.* Effects of tuberculosis, race, and human gene SLCO1B1 polymorphisms on rifampin concentrations *Antimicrob Agents Chemother* 2010 54 4192 4200 20660695 10.1128/AAC.00353-10 PMC2944564

[26] Cheli S Fusi M De Silvestri A Bonini I Clementi E Cattaneo D *et al.* In linezolid underexposure, pharmacogenetics matters: the role of CYP3A5 *Biomed Pharmacother* 2021 139 111631 33940510 10.1016/j.biopha.2021.111631

[27] Haas DW Abdelwahab MT van Beek SW Baker P Maartens G Bradford Y *et al.* Pharmacogenetics of between-individual variability in plasma clearance of bedaquiline and clofazimine in South Africa *J Infect Dis* 2022 226 147 156 35091749 10.1093/infdis/jiac024 PMC9373148

[28] Donald PR Parkin DP Seifart HI Schaaf HS van Helden PD Werely CJ *et al.* The influence of dose and N-acetyltransferase-2 (NAT2) genotype and phenotype on the pharmacokinetics and pharmacodynamics of isoniazid *Eur J Clin Pharmacol* 2007 63 633 639 17505821 10.1007/s00228-007-0305-5

[29] Azuma J Ohno M Kubota R Yokota S Nagai T Tsuyuguchi K *et al.* Pharmacogenetics-based tuberculosis therapy research group NAT2 genotype guided regimen reduces isoniazid-induced liver injury and early treatment failure in the 6-month four-drug standard treatment of tuberculosis: a randomized controlled trial for pharmacogenetics-based therapy *Eur J Clin Pharmacol* 2013 69 1091 1101 23150149 10.1007/s00228-012-1429-9 PMC3641305

[30] Verma R da Silva KE Rockwood N Wasmann RE Yende N A nanopore sequencing-based pharmacogenomic panel to personalize tuberculosis drug dosing [preprint] 2023 https://www.medrxiv.org/content/10.1101/2023.09.08.23295248v1 10.1164/rccm.202309-1583OC PMC11208962 38647526

[31] Verma R da Silva KE Rockwood N Wasmann RE Yende N Development and validation of a targeted nanopore sequencing panel to personalise treatment dosing in TB patients *Int J Tuberc Lung Dis* 2023 27 Suppl. 1

[32] Auton A Brooks LD Durbin RM Garrison EP Kang HM Korbel JO *et al.* The 1000 Genomes Project Consortium A global reference for human genetic variation *Nature* 2015 526 68 74 26432245 10.1038/nature15393 PMC4750478

[33] Rockwood N Meintjes G Chirehwa M Wiesner L McIlleron H Wilkinson RJ *et al.* HIV-1 coinfection does not reduce exposure to rifampin, isoniazid, and pyrazinamide in South African tuberculosis outpatients *Antimicrob Agents Chemother* 2016 60 6050 6059 27480859 10.1128/AAC.00480-16 PMC5038257

[34] WHO consolidated guidelines on drug-resistant tuberculosis treatment Geneva, Switzerland World Health Organization 2022 30946559

[35] Rockwood N Pasipanodya JG Denti P Sirgel F Lesosky M Gumbo T *et al.* Concentration-dependent antagonism and culture conversion in pulmonary tuberculosis *Clin Infect Dis* 2017 64 1350 1359 28205671 10.1093/cid/cix158 PMC5411399

[36] Vagena E Fakis G Boukouvala S Arylamine N-acetyltransferases in prokaryotic and eukaryotic genomes: a survey of public databases *Curr Drug Metab* 2008 9 628 660 18781915 10.2174/138920008785821729

[37] Sim SC Ingelman-Sundberg M The Human Cytochrome P450 (CYP) Allele Nomenclature website: a peer-reviewed database of CYP variants and their associated effects *Hum Genomics* 2010 4 278 281 20511141 10.1186/1479-7364-4-4-278 PMC3525213

[38] Ravichandran M Rajaram M Munusamy M Pharmacovigilance of antitubercular therapy in tuberculosis *Cureus* 2022 14 e21915 35273862 10.7759/cureus.21915 PMC8901153

[39] Rens NE Uyl-de Groot CA Goldhaber-Fiebert JD Croda J Andrews JR Cost-effectiveness of a pharmacogenomic test for stratified isoniazid dosing in treatment of active tuberculosis *Clin Infect Dis* 2020 71 3136 3143 31905381 10.1093/cid/ciz1212 PMC7819527

[40] Alwi ZB The use of SNPs in pharmacogenomics studies *Malays J Med Sci* 2005 12 4 12 22605952 PMC3349395

[41] Sabbagh A Langaney A Darlu P Gérard N Krishnamoorthy R Poloni ES Worldwide distribution of NAT2 diversity: implications for NAT2 evolutionary history *BMC Genet* 2008 9 21 18304320 10.1186/1471-2156-9-21 PMC2292740

[42] Sabbagh A Darlu P Vidaud M Evaluating NAT2PRED for inferring the individual acetylation status from unphased genotype data *BMC Med Genet* 2009 10 148 20043821 10.1186/1471-2350-10-148 PMC2806877

[43] Mthiyane T Millard J Adamson J Balakrishna Y Connolly C Owen A *et al.* *N*-acetyltransferase 2 genotypes among Zulu-speaking South Africans and isoniazid and *N*-acetyl-isoniazid pharmacokinetics during antituberculosis treatment *Antimicrob Agents Chemother* 2020 64 e02376 e19 31964788 10.1128/AAC.02376-19 PMC7179278

[44] Verma R Patil S Zhang N Moreira FMF Vitorio MT Santos ADS *et al.* A rapid pharmacogenomic assay to detect *NAT2* polymorphisms and guide isoniazid dosing for tuberculosis treatment *Am J Respir Crit Care Med* 2021 204 1317 1326 34375564 10.1164/rccm.202103-0564OC

[45] Nakajima A Fukami T Kobayashi Y Watanabe A Nakajima M Yokoi T Human arylacetamide deacetylase is responsible for deacetylation of rifamycins: rifampicin, rifabutin, and rifapentine *Biochem Pharmacol* 2011 82 1747 1756 21856291 10.1016/j.bcp.2011.08.003

[46] Francis J Zvada SP Denti P Hatherill M Charalambous S Mungofa S *et al.* A population pharmacokinetic analysis shows that arylacetamide deacetylase (AADAC) gene polymorphism and HIV infection affect the exposure of rifapentine *Antimicrob Agents Chemother* 2019 63 e01964-18 30670438 10.1128/AAC.01964-18 PMC6437540

[47] Paton NI Cousins C Suresh C Burhan E Chew KL Dalay VB *et al.* for the TRUNCATE-TB Trial Team Treatment strategy for rifampin-susceptible tuberculosis *N Engl J Med* 2023 388 873 887 36808186 10.1056/NEJMoa2212537 PMC7616851

[48] Conradie F Bagdasaryan TR Borisov S Howell P Mikiashvili L Ngubane N *et al.* ZeNix Trial Team Bedaquiline-pretomanid-linezolid regimens for drug-resistant tuberculosis *N Engl J Med* 2022 387 810 823 36053506 10.1056/NEJMoa2119430 PMC9490302

[49] Gorzynski JE Goenka SD Shafin K Jensen TD Fisk DG Grove ME *et al.* Ultrarapid nanopore genome sequencing in a critical care setting *N Engl J Med* 2022 386 700 702 35020984 10.1056/NEJMc2112090

[50] Mariner-Llicer C Goig GA Zaragoza-Infante L Torres-Puente M Villamayor L Navarro D *et al.* Accuracy of an amplicon-sequencing nanopore approach to identify variants in tuberculosis drug-resistance-associated genes *Microb Genom* 2021 7 000740 34919513 10.1099/mgen.0.000740 PMC8767343

[51] Xu Y Lewandowski K Lumley S Pullan S Vipond R Carroll M *et al.* Detection of viral pathogens with multiplex nanopore MinION sequencing: be careful with cross-talk *Front Microbiol* 2018 9 2225 30283430 10.3389/fmicb.2018.02225 PMC6156371

[52] Schmidt K Mwaigwisya S Crossman LC Doumith M Munroe D Pires C *et al.* Identification of bacterial pathogens and antimicrobial resistance directly from clinical urines by nanopore-based metagenomic sequencing *J Antimicrob Chemother* 2017 72 104 114 27667325 10.1093/jac/dkw397

